# Is gingival bleeding a symptom of type 2 and 3 von Willebrand disease?

**DOI:** 10.1371/journal.pone.0191291

**Published:** 2018-01-25

**Authors:** Lisa Epping, Wolfgang Miesbach, Katrin Nickles, Peter Eickholz

**Affiliations:** 1 Department of Periodontology, Center for Dentistry and Oral Medicine (Carolinum), Johann Wolfgang Goethe-University Frankfurt/Main, Frankfurt/Main, Germany; 2 Department of Haemostaseology/Haemophilia Centre, Medical Clinic II/Institute for Transfusion Medicine, University Hospital Frankfurt, Frankfurt/Main, Germany; Institut d'Investigacions Biomediques de Barcelona, SPAIN

## Abstract

**Background:**

Von Willebrand disease (VWD) is the most common inherent bleeding disorder. Gingival bleeding is a frequently reported symptom of VWD. However, gingival bleeding is also a leading symptom of plaque-induced gingivitis and untreated periodontal disease. In type 1 VWD gingival bleeding was not increased compared to controls. Thus, this study evaluated whether type 2 and 3 VWD determines an increased susceptibility to gingival bleeding in response to the oral biofilm.

**Methods:**

Twenty-four cases and 24 controls matched for age, sex, periodontal diagnosis, number of teeth and smoking were examined hematologically (VWF antigen, VWF activity, factor VIII activity) and periodontally (Gingival Bleeding Index [GBI]), bleeding on probing [BOP], Plaque Control Record [PCR], periodontal inflamed surface area [PISA], vertical probing attachment level).

**Results:**

BOP (VWD: 14.5±10.1%; controls: 12.3±5.3%; *p* = 0.542) and GBI (VWD: 10.5±9.9%; controls: 8.8±4.8%; *p* = 0.852) were similar for VWD and controls. Multiple regressions identified female sex, HbA1c, PCR and PISA to be associated with BOP. HbA1c and PCR were associated with GBI. Number of remaining teeth was negatively correlated with BOP and GBI.

**Conclusion:**

Type 2 and 3 VWD are not associated with a more pronounced inflammatory response to the oral biofilm in terms of BOP and GBI.

## Introduction

Von Willebrand disease (VWD) is the most common inherent bleeding disorder [1]. The disease is caused by deficiency or dysfunction of von Willebrand factor (VWF), a plasma protein that mediates platelet hemostatic function and stabilizes blood coagulation factor VIII. VWF is also the carrier of factor VIII in plasma. Thus, its deficiency may also result in low levels of factor VIII.

Estimates for prevalence of VWD range between 0.6 and 1.3% [2]. Inherent VWD is classified into three main types (1, 2 and 3) with type 2 divided into four subtypes (A, B, M, N) [3]. The most prevalent and mildest form is VWD type 1 (about 75%) representing a partial quantitative deficiency of VWF [1]. VWD type 2 (qualitative defects of VWF) accounts for around 20 to 25% of cases [1]. Subtype 2A exhibits impaired VWF multimer assembly (group 1 mutations) or increased proteolysis of VWF in circulation (group 2 mutations). Subtype 2B shows increased, type 2M decreased affinity of VWF for platelet GPIb receptor. Subtype 2N is characterized by decreased factor VIII because of VWF deficiently binding to factor VIII [1]. VWD type 3 (0.6 to 6% of cases) represents complete quantitative deficiency of VWF [1, 3, 4]. These three major types of VWD may affect both males and females. Type 1 and type 2A and 2B are inherited in an autosomal dominant manner while type 2M, 2N and 3 are inherited in an autosomal recessive manner [3].

People with VWD bruise easily, have recurrent mucocutaneous bleeding, or bleed after tooth extraction, tonsillectomy or other surgery [5]. A common symptom of VWD is epistaxis (nose bleeding). Furthermore, women can have increased menstrual bleeding. Also a frequently reported symptom of VWD is gingival bleeding [6–8].

Gingival bleeding is also a leading symptom of plaque-induced gingivitis and untreated periodontal disease [4, 9, 10]. Interestingly case reports on VWD reporting spontaneous gingival bleeding as symptom did not look into periodontal health in detail. Abbas & Prabhu report “fair oral hygiene” and the gingival tissues to “look otherwise healthy” [6]. In another case continual bleeding 12 hours after scaling and polishing of the lower teeth is reported [8]. However, plaque indices or periodontal variables as probing pocket depths (PPD) and vertical probing attachment level (PAL-V) [6, 8]. A case control study comparing type 1 VWD with controls matched for sex, age, number of teeth, severity of periodontal disease, and smoking failed to detect increased gingival bleeding in VWD [4].

## Material & methods

### Patients

In the present prospective case control study patients suffering from type 2 and 3 VWD (VWF Ristocetin cofactor [VWF:RCo] < 30%) were compared to hematologically healthy controls matched for sex, age, number of teeth, severity of periodontal disease, and smoking.

This study applies the protocol of a case control study comparing type 1 VWD with controls matched for sex, age, number of teeth, severity of periodontal disease, and smoking to type 2 and 3 VWD patients [4]. All patients with type 2 and 3 VWD consecutively consulting the Haemophilia Centre, Medical Clinic II/Institute for Transfusion medicine, Hospital of the Johann Wolfgang Goethe-University Frankfurt/Main were asked to participate in this study as cases. They were asked for bleeding and subjective symptoms indicating periodontal disease. This study is a human observational study and conforms to the STROBE guidelines.

The study complied with the rules of the Declaration of Helsinki and was approved by the Institutional Review Board for Human Studies of the Medical Faculty of the Goethe-University Frankfurt/Main (Application# 143/15). All participating individuals were informed on risks and benefits as well as the procedures of the study and gave written informed consent. The study is registered under the number NCT03078595 at http://www.clinicaltrials.gov.

### Inclusion criteria

- Age between 18 and 80 years,
- Written informed consent,
- Formerly diagnosed type 2 and 3 VWD (according to VWF multimer analysis and VWF:RCo < 30%)

### Exclusion criteria

- Requirement of systemic antibiotics for measures that may cause transitory bacteremia (e.g. pocket probing),
- VWD type 1 (VWF:RCo > 30%),
- Additional bleeding disorders (e.g. hemophilia A or B),
- Anticoagulation or antiplatelet treatment (e.g. acetylsalicylic acid, warfarin)

### Controls

For each case (VWD) a respective hematologically healthy control was recruited from the gingivitis and periodontitis patients of the Department of Periodontology, Center for Dentistry and Oral Medicine (Carolinum), Johann Wolfgang Goethe-University Frankfurt/Main. Each control was matched to one of the respective cases for sex, age (±5 years), self-reported smoking status (current smoker/non-smoker), number of remaining teeth (±2 teeth), and periodontal diagnosis (gingivitis, chronic or aggressive periodontitis).

Smoking may interfere with gingival bleeding [11, 12]. Thus, all participants were asked about current and past cigarette smoking habits. Patients who reported smoking or had quit smoking for less than five years were classified as smokers [13]. Additionally the amount of carbon monoxide (CO) in exhaled air was measured using a Smokerlyzer^®^ (Bedfont Smokerlyzer EC50-Micro; Bedfont Scientific Ltd, Rochester, Great Britain).

### Hematologic examinations

Twenty ml of blood was sampled from an arm vein. The following data were assessed at the Haemophilia Centre for clinical routine during VWD patient care and due to study design as well as to determine whether haemotological disease was present or not in the controls:

- von Willebrand parameters (VWF antigen [VWF:Ag], VWF activity [VWF:Act], coagulation factor VIII [FVIII:C])
- Current medication if any

Also diabetes mellitus and glycemic control may interfere with systemic as well as periodontal inflammation and, hence, gingival bleeding [14, 15]. Even so called prediabetes may contribute to inflammation. However, the state of prediabetes may not be known by the affected individual and not detected by medical history [15]. Thus, HbA1c as a measure for glycemic control was assessed also.

VWF:Ag and VWF:Act were measured turbidimetrically using a BCS (Siemens, Marburg, Germany). FVIII:C was assessed with specific agents on a coagulation analyzer (ACL-700^®^, IL Instrumentation Laboratory, Kirchheim, Germany).

### Periodontal examinations

For all participants, a commercially available test (PerioSafe^®^, Dentagnostics, Jena, Germany) was performed to detect activated matrix metalloproteinase 8 (aMMP-8) from the gingival sulcus. First, patients rinsed with tap water for 30 seconds then spat the water out. After 1 minute, the patients rinsed with 5mL of purified water for 30 seconds and spat the sample back into the test cup. A syringe was used to gather 2mL of the sampled saliva and water mixture. A filter was then put onto the syringe and 3 drops of the saliva was pressed through the filter into the ELISA kit. After 5–10 minutes, the result was read from the test kit [16]. If both the control and test strips were visible (≥ 25 ng aMMP 8 per mL), the respective test was positive.

The following clinical parameters were assessed at 6 sites per tooth (mesiobuccal, buccal, distobuccal, mesiolingual, lingual, distolingual) [4]:

- modified Gingival Bleeding Index (GBI) [17]
- modified Plaque Control Record (PCR) [18]
- PPD and recession to the nearest 0.2 mm using an electronic probe (Florida Probe, Version 3.2, Gainesville, FL, USA). Recession was assessed from the cemento-enamel junction (CEJ) to the gingival margin. At sites where the CEJ was destroyed by restorations the restoration margin (RM) was used as reference. At sites where the CEJ or RM was located apically from the gingival margin the value for recession was negative
- Bleeding on probing (BOP) recorded as positive when bleeding occurred within 30 seconds from probing. For each patient a BOP index was calculated providing the amount of sites with positive BOP in % per patient.
- Attachment loss was calculated as sum of PPD and recession. At sites where the gingival margin was located coronally of the CEJ recession was scored as a negative value.

All individuals were classified into the following diagnoses [14, 19]:

- plaque-induced gingivitis (PPD < 3.6 mm; PAL-V ≤ 2 mm),
- generalized mild, localized moderate chronic periodontitis (PPD ≥ 3.6 mm; PAL-V 3 to 4 mm ≤ 30% of sites; 1 to 2 mm > 30% of sites),
- generalized mild, localized severe chronic periodontitis (PPD ≥ 3.6 mm; PAL-V ≥ 5 mm ≤ 30% of sites; 1 to 2 mm > 30% of sites)
- generalized moderate chronic periodontitis (PPD ≥ 3.6 mm; PAL-V 3 to 4 mm > 30%)
- generalized moderate localized severe chronic periodontitis (PPD ≥ 3.6 mm; PAL-V 3 to 4 mm > 30%; ≥ 5 mm ≤ 30%)

In all individuals hematological and periodontal examinations were obtained within 24 hours. After dental and periodontal examination all patients received oral hygiene instructions and professional tooth cleaning. In cases of untreated periodontal disease periodontal treatment was offered. Increased bleeding may occur at home after patients had already left the clinic. Thus, only subjects with VWD were asked to report any bleeding complications after periodontal probing and professional tooth cleaning.

### Statistical analysis

The individual patient was used as statistical unit. All analyses were performed on patient level. BOP was defined as the main outcome variable and GBI as secondary outcome variable. All other parameters were control variables. A sample size of n = 62 (31 VWD cases and 31 controls) was required to detect an inter-group difference of 5.5% [4] GBI or BOP with a type 1 error α < 0.05 and a test power of 80% (http://jumbo.uni-muenster.de/fileadmin/jumbo/applets/falla.html). After inclusion of 24 type 2 and 3 VWD cases no further type 2 and 3 VWD cases could be recruited at the Haemophilia Centre, Medical Clinic III/Institute for Transfusion medicine, Hospital of the Johann Wolfgang Goethe-University Frankfurt/Main. Thus, the study was analyses with 24 cases and 24 matched controls.

For all individuals, cigarette pack years were calculated. Group frequencies (VWD, control) were expressed for sex, current smoking. Group means and standard deviations were calculated for GBI, BOP, age, number of remaining teeth, pack years, CO, PCR, Body weight, VWF:Ag, VWF:RCO, FVIII:C, Hb1c. Further, for each individual the following variables were calculated to describe the periodontal status:

- Mean±standard deviation of PPD and PAL-V
- Percentage of PPD < 4 mm, 4 to 6.8 mm, ≥ 7 mm
- Sum of all PPD (Wohlfeil et al. 2009), i.e. the sum the PPD measured at all sites within a patient
- Sum of all PPD with BOP (Wohlfeil et al. 2009), i.e. the sum the PPD measured at all sites exhibiting BOP within a patient
- Periodontal inflamed surface area (PISA) [20, 21]. For each patient PPD were entered into an Excel sheet that can be downloaded freely (http://www.parsprototo.info/pisa.html).

From these group means and standard deviations were calculated. Comparisons between groups for dichotomous parameters were made by χ^2^ or Fisher’s exact test and for all other parameters by Mann-Whitney-U test. A post-hoc analysis was performed to estimate the test power that would be required to find a clinically relevant inter-group difference (δ) of 5.5% for BOP and GBI index with a type 1 error (α) of 0.05 for the actual sample size.

Using stepwise linear backward multiple regression analysis, factors should be identified that influenced BOP and GBI. The following independent variables were entered into the model for BOP: group (VWD/control), VWD type 2 or 3, sex, age, body weight, HbA1c, PCR, CO, aMMP-8, number of remaining teeth, PISA. The following independent variables were entered into the model for GBI: group (VWD/control), VWD type 2 or 3, sex, age, body weight, HbA1c, PCR, CO, aMMP-8, number of remaining teeth, PISA. Due to the fact that mean PPD is mathematically coupled to sum of PPD, sum of PPD with BOP, and PISA these 4 variables were not entered into the regression model at the same time. PISA provides the best representation of the subgingival inflamed area. Thus, PISA was chosen for the final model. The following parameters were described by dummy variables: group (control = 0, VWD = 1), sex (male = 0, female = 1), smoking status (never and former smoker = 0, current smoker = 1). All factors with *p* < 0.05 were kept in the models. For statistical analysis a PC program was used (Systat^TM^ for Windows Version 13, Systat Inc., Evanston, USA).

## Results

At the Haemophilia Centre, Medical Clinic III/Institute for Transfusion Medicine, Hospital of the Johann Wolfgang Goethe-University Frankfurt/Main approximately 1500 charts of VWD patients were screened rendering about 35 patients with VWD type 2 and 3. From July 16, 2015 to July 15, 2016 24 type 2 and 3 VWD cases were enrolled. Due to the difficulty to find more type 2 and 3 VWD patients willing to participate recruitment of cases was stopped in March 2017 after enrolment of 24 individuals. From April 04, 2016 to March 24, 2017 24 patients that self-reported to be hematologically healthy were proven to be hematologically healthy, periodontally examined and matched to respective VWD cases. Of the 20 type 2 VWD patients 11 were subtype A, 4 subtype B, 3 M, and 2 N. Four were type 3 VWD. 24 controls were enrolled. Patient characteristics of cases and controls are provided by Table 1. Both groups are well balanced according to sex, age, HbA1c, nicotine consumption (self-report and assessed as CO in exhaled air), number of remaining teeth, and periodontal diagnosis (Table 1). CO and self-reported smoking status (r^2^ = 0.73, *p* < 0.001) as well as pack years (r^2^ = 0.71, *p* < 0.001) were strongly correlated. CO was significantly higher in self-reported smokers (8.7±3.7 ppm) than in non-smokers (1.2±0.8 ppm) (*p* < 0.001). Due to case definition VWF:Ag, VWF:RCO, and FVIII:C are significantly lower in VWD than in controls (Table 2).

**Table 1:** Patient characteristic (chronic periodontitis: ChP).

	Cases (VWD) N = 24	Controls N = 24	*p*
Female sex [n/%]	17/71%	17/71%	
Age [years]	45.9±11.8	46.6±12.4	
Number of teeth [n]	26.7±2.9	26.8±2.2	
Current smokers [n/%]	7/29%	7/29%	
Exhaled CO [ppm]	3.9±4.6	2.8±3.4	0.300
Pack years [n]	3.3±6.4	1.6±2.9	0.598
Body weight [kg]	71.6±16.1	68.5±9.8	0.757
HbA1c [%]	5.1±0.6	5.0±0.4	0.975
Diagnoses			
Gingivitis [n/%]	11/45.8%	11/45.8%	
Generalized mild, localized moderate ChP [n/%]	5/20.8%	5/20.8%	
Generalized mild, localized severe ChP [n/%]	3/12.5%	3/12.5%	
Generalized moderate ChP [n/%]	2/8.4%	2/8.4%	
Generalized moderate localized severe ChP [n/%]	3/12.5%	3/12.5%	

**Table 2:** Hematologic variables.

	Cases (VWD) N = 24	Controls N = 24	*p*
Von Willebrand antigen [%] (normal range: 60–200%)	44.0±45.9	122.1±47.2	<0.001
Von Willebrand activity [%] (normal range: 60–200%)	24.7±23.4	122.1±45.1	<0.001
Factor VIII [%] (normal range: 68–133%)	55.6±46.3	129.8±27.5	<0.001

Cases and controls were well balanced regarding periodontal control variables (PCR, PPD, PAL-V) (Tab. 3). The study failed to find any significant difference regarding BOP and GBI between VWD (14.5%/10.5%) and controls (12.3%/8.8%) (Table 3). The actual inter-group difference of 2.2% for BOP index (standard deviation 10.1%) can be shown with a type 1 error (α) of 0.05 and a test power of 12% with 24 patients in each group. The respective test power for GBI (inter-group difference 1.7%; standard deviation 9.9%) is 9%. None of the VWD reported any bleeding complications after periodontal probing and professional tooth cleaning.

**Table 3:** Periodontal variables (probing pocket depth: PPD; vertical probing attachment level: PAL-V).

	Cases (VWD) N = 24	Controls N = 24	*p*
Bleeding on Probing [%]	14.5±10.1	12.3±5.3	0.542
Gingival Bleeding Index [%]	10.5±9.9	8.8±4.8	0.852
Plaque Control Record [%]	53.0±24.1	49.5±15.9	0.820
Activated metallomatrix proteinase 8 [n/%]	8/33%	8/33%	1.000
Mean PPD [mm]	1.8±0.6	1.9±0.5	0.470
Mean PAL-V [mm]	1.9±0.7	1.9±0.6	0.509
Sum of PPD [mm]	295.0±102.6	306.7±87.6	0.273
Sum of PPD with BOP [mm]	54.4±37.8	49.7±28.1	0.926
Periodontal inflamed surface area [mm^2^]	154.4±124.0	136.1±95.3	0.665
PPD < 4 mm [%]	98.5±3.8	98.7±3.4	0.812
PPD 4 to 6.8 mm [%]	1.5±3.8	1.3±3.4	0.812
PPD ≥ 7 mm [%]	0.0±0.0	0.0±0.0	1.000

Multiple regression identified female sex (*p* = 0.001), HbA1c (*p* < 0.001), PCR (*p* < 0.001) and PISA (*p* = 0.015) to be associated with BOP (Table 4). Further, multiple regression identified HbA1c (*p* = 0.010) and PCR (*p* < 0.001) to be associated with GBI (Table 5). Number of remaining teeth was negatively correlated with BOP and GBI.

**Table 4:** Backward stepwise multiple regression analysis: Bleeding on Probing in relation to individual and periodontal variables.

Dependent variable: Bleeding on probing Index; n = 48; R^2^ = 0.768; R^2^adjusted = 0.740; standard error of estimate = 4.112				
	*b*	s.e.(*b*)	T	*p*
Constant	-15.528	10.863	-1.429	0.160
HbA1c	6.053	1.398	4.329	< 0.001
Female sex	4.618	1.352	3.415	0.001
Plaque Control Record	0.190	0.036	5.268	< 0.001
Periodontal inflamed surface area	0.017	0.007	2.547	0.015
Number of remaining teeth	-0.647	0.246	-2.628	0.012
Analysis of variance: *p* < 0.001				

**Table 5:** Backward stepwise multiple regression analysis: Gingival Bleeding Index in relation to individual and periodontal variables.

Dependent variable: Gingival Bleeding Index; n = 48; R^2^ = 0.617; R^2^adjusted = 0.591; standard error of estimate = 4.958				
	*b*	s.e.(*b*)	T	*p*
Constant	11.027	12.436	0.887	0.380
HbA1c	4.380	1.623	2.699	0.010
Plaque Control Record	0.172	0.037	4.589	< 0.001
Number of remaining teeth	-1.212	0.295	-4.106	< 0.001
Analysis of variance: *p* < 0.001				

## Discussion

A frequently reported symptom of VWD is gingival bleeding [6–8]. A previous case control study comparing type 1 VWD with controls matched for sex, age, number of teeth, severity of periodontal disease, and smoking failed to detect increased gingival bleeding (GBI, BOP) in VWD [4]. Expecting more severe bleeding with the more severe VWD type 2 and 3 the actual similar case control study was initiated. However, even comparing type 2 and 3 VWD with controls matched for sex, age, number of teeth, severity of periodontal disease, and smoking this study failed to detect increased gingival bleeding (GBI, BOP) in VWD. This is in line with a study in 44 female VWD cases in which plaque and dental care utilization was found to determine gingival bleeding more than the levels of VWF (i.e. severity of VWD) [22].

Besides hemostasis several factors may affect gingival/periodontal bleeding. Females have been shown to exhibit higher BOP frequencies than comparable males [12]. This study revealed an association between GBI and female sex. Current smoking is another important factor that may interfere with gingival inflammation [11, 12]. However, there are several variables to assess smoking (e.g. self-report of patients, cotinine levels in urine, CO in exhaled air). Thus, CO in exhaled air was assessed additionally to self-report in this study to increase objectivity and cover the effect of second-hand smoking also. Self-reported smoking status and CO correlated well in this study confirming the reliability of self-report [4, 23, 24]. However, none of the smoking variables could be associated with bleeding.

In addition to the analyses in a previous study, for this sample serum HbA1c levels were assessed. HbA1c is a measure for glycemic control. Diabetes mellitus, prediabetes and glycemic control may affect periodontal inflammation and, hence, gingival bleeding [14, 15]. Hb1Ac was strongly associated with BOP and GBI confirming the significant role glycemic control plays with regard to periodontal inflammation. Number of remaining teeth was associated with less inflammation (BOP and GBI). This confirms results of a previous study regarding GBI [4]. More remaining teeth may be interpreted as less periodontal disease due to a less severe inflammatory response.

BOP and periodontal pocketing are symptoms of subgingival inflammation. Thus, various variables were assessed to measure the degree of periodontal disease: mean PPD and PAL-V, sum of all PPD [25] as well as sum of PPD with BOP per patient and PISA [20, 21]. Other groups have demonstrated that the probability of BOP correlates with the number of deep pockets [12, 26]. The actual study confirmed these results by finding an association between BOP and PISA. Thus, VWD and controls were matched according to sex, age, number of teeth, severity of periodontal disease, and smoking to control for the respective factors.

Gingival bleeding is a leading symptom of plaque-induced gingivitis [9, 10] and untreated periodontal disease [27]. In those cases that gingival bleeding is reported as symptom of VWD information on periodontal health or disease (plaque indices, PPD, PAL-V) is not provided [4, 6, 8]. The amount of supragingival plaque (i.e. PCR) was positively associated with gingival inflammation in type 1 VWD [4] and in this study also in type 2 and 3 VWD. Thus, gingival bleeding in VWD is likely to be due to dental biofilm and periodontal disease and not a genuine symptom caused by this bleeding disorder.

Regarding the actual inter-group difference of 2.2% for BOP index (standard deviation 10.1%) between VWD and controls this study is underpowered (test power of 12% with a type 1 error of 0.05 with 24 patients in each group). However, 2.2% more BOP in type 2 and 3 VWD compared to hematologically healthy controls is a quite small and clinically irrelevant difference.

As in the recent study [4] type 2 and 3 VWD and controls included into this study suffered from mild to moderate periodontal disease and exhibited quite low BOP (14.5/12.3%) compared to untreated aggressive periodontitis and generalized severe ChP (48.6/55.9%) [27]. Thus, we may hypothesize that the difference in gingival bleeding between VWD and control patients would have become significant if patients with a greater severity of destructive periodontal disease had been compared.

Subgingival scaling and periodontal surgery in VWD patients may cause prolonged bleeding and therefore requires certain measures to prevent bleeding complications that are expensive and exhausting to patients [28]. Thus, VWD patients may significantly benefit from prevention of periodontal disease. Even in type 2 and 3 VWD periodontal examination and professional tooth cleaning may be rendered without increased risk of bleeding.

Despite being underpowered what are the limitations of this study? A future study may investigate the hypothesis that gingival bleeding may be more severe in VWD than in control individuals if patients with more severe periodontal disease are examined. However, it was difficult to recruit enough type 2 and 3 VWD patients at all. It will be more difficult to find those with more severe periodontal disease. Another interesting question is how hemophilia A and B affect gingival bleeding. Perhaps these even more severe bleeding disorders may make a difference regarding gingival bleeding compared to hematologically healthy controls.

## Conclusions

Within the limitations of the present study the following conclusions may be drawn:

- Type 2 and 3 VWD is not associated with a more pronounced inflammatory response to the oral biofilm in terms of GBI and BOP.
- HbA1c is significantly associated with gingival bleeding.

## Supporting information

S1 Table Raw data. F1 to 12: questions 1 to 12 of questionnaire*; VWDTYP: 0 (control), 1 (VWD type 2), 2 (VWD type 3); VWD2TYP: subtypes of VWD type 2; HBA1C: % glycated hemoglobin A1c; VWFAG: von Willebrand factor antigen; RICO: VWF activity; FVIII: coagulation factor VIII; BLUTGRUP: blood group; HPPB: hepatitis B infection: 0 (no), yes (1)*; HEPC: hepatitis C infection: 0 (no), yes (1)*; HIV human immunodeficiency virus infection: 0 (no), yes (1)*; SMOKER: current smokers: 0 (no), yes (1); RAUCHERE: current and former smokers: 0 (no), yes (1); PPM: ppm of CO in exhaled air; AMMP8: activated matrix metalloproteinase 8≥ 25 ng per mL: 0 (no), yes (1); BS: score of ISTH-BAT questionnaire *; MINBS: time for obtaining ISTH-BAT questionnaire in minutes*; NUMBER_TEETH: remaining teeth; DIAGNOSE: diagnosis: 0 (plaque-induced gingivitis), 1 (generalized mild, localized moderate chronic periodontitis), 2 (generalized mild, localized severe chronic periodontitis), 3 (generalized moderate chronic periodontitis), 4 (generalized moderate localized severe chronic periodontitis); TRANEX: tranexam acid required for examination/treatment: 0 (no), yes (1); SUMPD: sum of probing depth (PD) in mm; PD_MEAN: mean PD per patient; CAL_MEAN: mean clinical attachment level per patient; PD14: percentage of sites with PD < 4 mm; PD56: percentage of sites with PD 4 to 6.8 mm; PD7: percentage of sites with PD ≥ 7 mm; STBOP: sum of PD with bleeding in mm; CPD: cummulative PD (sum of all PD ≥ 4 mm)*; PISA: periodontally inflamed surface area in mm^2^; HAEM 1 to 14: questions 1 to 14 of ISTH-BAT-questionnaire*;
* not analysed in this study.
(PDF)
