# Supplementary material for: Is gingival bleeding a symptom of type 2 and 3 von Willebrand disease?
Source: PLoS One. 2018 Jan 25;13(1):e0191291. doi: 10.1371/journal.pone.0191291 (PMC5784962; doi:10.1371/journal.pone.0191291)
Supplement: S1 Table — F1 to 12: questions 1 to 12 of questionnaire*; VWDTYP: 0 (control), 1 (VWD type 2), 2 (VWD type 3); VWD2TYP: subtypes of VWD type 2; HBA1C: % glycated hemoglobin A1c; VWFAG: von Willebrand factor antigen; RICO: VWF activity; FVIII: coagulation factor VIII; BLUTGRUP: blood group; HPPB: hepatitis B infection: 0 (no), yes (1)*; HEPC: hepatitis C infection: 0 (no), yes (1)*; HIV human immunodeficiency virus infection: 0 (no), yes (1)*; SMOKER: current smokers: 0 (no), yes (1); RAUCHERE: current and former smokers: 0 (no), yes (1); PPM: ppm of CO in exhaled air; AMMP8: activated matrix metalloproteinase 8≥ 25 ng per mL: 0 (no), yes (1); BS: score of ISTH-BAT questionnaire *; MINBS: time for obtaining ISTH-BAT questionnaire in minutes*; NUMBER_TEETH: remaining teeth; DIAGNOSE: diagnosis: 0 (plaque-induced gingivitis), 1 (generalized mild, localized moderate chronic periodontitis), 2 (generalized mild, localized severe chronic periodontitis), 3 (generalized moderate chronic periodontitis), 4 (generalized moderate localized severe chronic periodontitis); TRANEX: tranexam acid required for examination/treatment: 0 (no), yes (1); SUMPD: sum of probing depth (PD) in mm; PD_MEAN: mean PD per patient; CAL_MEAN: mean clinical attachment level per patient; PD14: percentage of sites with PD < 4 mm; PD56: percentage of sites with PD 4 to 6.8 mm; PD7: percentage of sites with PD ≥ 7 mm; STBOP: sum of PD with bleeding in mm; CPD: cummulative PD (sum of all PD ≥ 4 mm)*; PISA: periodontally inflamed surface area in mm2; HAEM 1 to 14: questions 1 to 14 of ISTH-BAT-questionnaire*; * not analysed in this study. (PDF) [file pone.0191291.s001.pdf]

NUMBER	INITIALs	TEST	FEMALE	F11	VWDTYP	VWD2TYP
1	SEMA	1	1	0	1	A
2	STMO	1	1	1	1	N
3	MOHE	1	1	1	1	A
4	KAGE	1	0		1	A
5	MOSA	1	1	1	1	M
6	MOEL	1	1	1	1	M
7	ROLI	1	1	0	1	B
8	GUTH	1	0		1	N
9	MUAC	1	0		2	
10	PEOR	1	0		2	
11	PACA	1	1	1	1	A
12	AWAT	1	0		2	
13	VEMO	1	1	1	1	A
14	WIRO	1	0		1	B
15	FUIR	1	1	0	1	A
16	MOGR	1	0		1	B
17	HONA	1	1	1	1	A
18	MESA	1	1	1	1	A
19	WIAN	1	1	1	2	
20	KAHA	1	1	1	1	M
21	HOUL	1	1	1	1	A
22	SCDO	1	1	1	1	B
23	PFCH	1	1	1	1	A
24	GREL	1	1	1	1	A
1	HUEL	0	1	0	0	
2	KISO	0	1	0	0	
3	KLAN	0	1	0	0	
4	LAEV	0	1	0	0	
5	RATA	0	1	0	0	
6	CAMA	0	1	1	0	
7	JUTH	0	0		0	
8	GOHE	0	1	0	0	
9	MUSO	0	1	0	0	
10	EPST	0	0		0	
11	REHE	0	0		0	
12	FUMA	0	0		0	
13	VOSA	0	1	0	0	
14	WEHA	0	0		0	
15	STBR	0	1	0	0	
16	COER	0	1	0	0	
17	STIR	0	1	0	0	
18	BOCL	0	1	0	0	
19	GEBI	0	1	0	0	
20	YIAY	0	1	0	0	
21	SAJA	0	1	0	0	
22	HAIR	0	1	0	0	
23	ALTA	0	0		0	
24	VOFR	0	0		0	

AGE	WEIGHT	HBA1C	VWFAG	RICO	FVIII	BLUTGRUP
32	54	4,86	22,8	12,9	33,2	AB RH POS
62	63	5,27	79,8	83,7	74,1	O RH POS
51	65	5,35	42,5	13,6	36	A RH POS
56	68	5,25	19,4	59,7	36,3	AB RH POS
35	70	5,57	103	19,8	127,1	O RH POS
64	100	6,68	227,9	48	225,6	O RH POS
33	52	4,7	30	5	33,2	O RH NEG
43	86	4,7	34,3	38,5	17,2	A RH NEG
56	83	5,31	10	7,8	40,5	A RH NEG
49	90	5,23	10	4	2,4	O RH POS
50	69	5,27	19,2	13,1	52,1	A RH POS
40	108	6,44	10	4	4,5	0 RH POS
41	80	5,12	45	36,6	90,5	A RH POS
44	101	5,05	33	12,2	36,7	A RH POS
34	68	4,91	19	12,5	39,1	O RH POS
21	70	5,07	67	62,5	87,4	B RH POS
45	58	4,29	26	25,9	50,1	A RH POS
56	55	5,05	35	9	49	A RH NEG
52	68	4,62	9	4,2	7	A RH POS
26	56	4,78	24	11	54,6	ABPOS
40	53	4,11	67	69,9	75,6	0POS
65	55	4,92	46	10,5	58,9	0POS
50	68	4,66	52	15	63,4	0NEG
56	78	5,31	23	13,5	39,3	APOS
39	74	5,47	81	99	107,5	0POS
32	52	4,43	150	123,2	155,1	UNBEKANNT
33	84	5,21	110	91,5	116,4	UNBEKANNT
25	69	4,58	120	108	111,9	BPOS
42	55	4,6	110	97,1	122,2	BPOS
49	65	4,78	110	137,5	118,3	ABPOS
44	78	4,79	110	97,6	135,8	0NEG
32	60	4,91	120	117,9	101,7	APOS
58	50	4,95	100	119	100,1	0NEG
27	78	4,9	98	128	104,9	ANEG
62	65	5,37	83	83,7	107,5	0POS
49	76	5,21	170	154,8	188,5	UNBEKANNT
58	58	5,08	110	117,6	136,1	0POS
59	75	5,27	150	104,9	142,7	APOS
65	55	5,1	239	242,8	181,1	0POS
53	83	5,44	110	119,9	129	APOS
65	74	5,46	271	261,7	200,8	0POS
55	75	5,64	84	100,3	132	0POS
56	63	4,6	74	71	113,2	0POS
36	65	4,64	92	78,8	138,3	BPOS
30	77	4,71	88	95,8	124,1	APOS
55	76	5,45	130	142,6	129,9	BPOS
45	68	5,12	100	101	98,5	APOS
50	68	5,41	120	135,8	119,2	APOS

HEPB	HEPC	HIV	SMOKER	RAUCHERE	PPM	PACKYEAR
0	0	0	1	1	8	2
0	0	0	1	1	11	10
0	0	0	1	1	13	10
0	0	0	0	0	3	0
0	0	0	1	1	13	13
0	0	0	0	0	1	0
0	0	0	0	0	2	0
0	0	0	1	1	9	14
1	1	0	0	0	2	0
0	0	0	1	1	14	25
0	0	0	0	1	1	2
0	1	0	0	0	1	0
0	0	0	0	0	1	0
0	0	0	0	0	0	0
0	0	0	0	0	0	0
0	0	0	0	0	2	0
0	0	0	1	1	4	2
0	1	0	0	0	2	0
0	1	0	0	0	1	0
0	0	0	0	0	1	0
0	0	0	0	0	1	0
0	0	0	0	0	1	0
0	0	0	0	0	2	0
0	0	0	0	0	1	0
0	0	0	0	0	1	0
0	0	0	0	1	2	0
0	0	0	0	0	1	0
0	0	0	0	0	2	0
0	0	0	0	0	1	0
0	0	0	0	0	0	0
0	0	0	0	0	1	0
0	0	0	1	1	11	5
0	0	0	0	1	3	0
0	0	0	0	0	1	0
0	0	0	0	0	1	0
0	0	0	1	1	8	7
0	0	0	0	0	1	0
0	0	0	0	0	0	0
0	0	0	0	0	2	0
0	0	0	0	0	0	0
0	0	0	0	0	0	0
0	0	0	1	1	1	5
0	0	0	0	0	1	0
0	0	0	1	1	6	5
0	0	0	1	1	7	10
0	0	0	1	1	11	5
0	0	0	0	0	1	0
0	0	0	1	1	6	1,5

AMMP8	F1	F2	F2A	F3	F4	F5
0	0	1	0	0	0	0
0	1	0	0	0	0	1
0	1	1	0	0	0	1
0	0	0	1	1	0	1
1	0	1	0	1	0	1
1	0	0	1	1	1	0
0	0	1	0	0	1	1
0	0	1	1	0	0	0
0	0	0	1	1	0	0
1	0	0	1	1	1	0
0	0	0	1	1	0	1
0	0	0	1	1	0	0
1	0	0	1	0	0	0
0	1	1	1	0	0	1
1	0	0	0	1	0	0
1	0	0	0	1	1	0
0	1	1	0	0	1	1
0	1	0	0	0	0	1
0	0	0	1	1	0	0
0	0	1	0	0	0	0
1	0	0	0	1	1	0
1	0	1	1	1	1	0
0	0	1	1	1	1	0
0	0	0	0	1	0	0
0	0	1	0	0	0	0
0	0	1	0	0	0	0
1	0	1	0	0	0	0
0	0	1	0	0	0	0
0	0	1	0	0	0	0
0	0	1	0	0	0	0
1	0	0	0	1	1	0
0	0	1	0	0	0	0
1	0	1	0	0	0	0
1	0	1	0	0	0	0
1	0	0	0	1	1	0
1	0	0	0	1	1	0
0	1	1	0	0	0	1
1	1	1	0	0	0	0
0	0	1	0	0	0	0
0	0	1	0	0	0	0
0	1	1	0	0	0	0
0	0	1	0	0	0	0
0	0	1	0	0	0	0
1	1	0	0	0	0	0
0	0	1	0	0	0	0
0	1	1	0	0	0	1
0	0	1	0	0	0	0
0	0	1	0	0	0	0

F6	F7	F8	F9	F10	F12	BS
0	0	1	1	1	1	18
0	1	1	1	1	0	19
1	0	1	1	1	1	40
0	0	1	0	1	1	6
0	0	1	0	1	1	14
1	1	1	1	1	1	28
0	0	1	1	1	1	26
0	0	1	1	1	0	25
0	0	1	1	1	1	29
0	0	1	1	1	1	19
0	1	1	1	1	1	16
0	0	1	1	1	1	17
0	0	0	1	1	1	17
0	0	1	1	1	1	14
0	0	1	1	1	1	12
0	1	1	1	1	1	13
1	0	1	1	1	0	15
1	0	1	1	1	1	20
0	0	1	1	1	1	37
0	0	1	1	1	1	12
0	0	1	1	1	1	19
0	0	1	1	1	1	29
0	0	1	1	1	1	18
0	0	1	1	1	0	24
0	0	0	0	0	0	6
0	0	0	0	0	0	3
0	0	0	0	0	0	2
0	0	0	0	0	0	5
0	0	0	0	0	0	1
0	0	1	0	0	0	7
0	0	0	0	0	0	1
0	0	0	0	0	0	3
0	0	0	0	0	0	4
0	0	0	0	0	0	2
0	0	0	0	0	0	1
0	0	0	0	0	0	2
0	0	0	0	0	0	3
0	0	0	0	0	0	0
0	0	0	0	0	0	0
0	0	0	0	0	0	1
0	0	0	0	0	0	0
0	0	0	0	0	0	2
0	0	1	0	0	0	1
0	1	0	0	0	0	0
0	0	0	0	0	0	1
0	0	0	0	0	0	3
0	0	0	0	0	0	0
0	0	0	0	0	0	1

MINBS	JMBER_TEETH	DIAGNOSE	GBI	PCR	BOP	TRANEX
17	28	0	2	26	7	0
25	23	2	10	49	12	0
33	27	4	16	39	20	0
13	20	2	11	31	6	0
25	27	1	15	81	25	0
35	20	1	52	100	53	0
27	28	0	2	20	1	0
18	27	0	4	37	5	0
35	27	0	8	28	6	0
30	27	4	8	100	20	0
37	26	1	8	33	11	0
32	27	0	9	57	18	1
38	26	3	10	74	19	0
26	28	2	5	57	10	0
24	31	0	6	58	12	0
20	30	0	15	83	18	0
25	21	1	20	54	21	0
35	29	1	5	54	14	0
43	27	0	5	26	14	0
25	28	0	5	45	7	0
22	27	0	10	68	8	0
27	28	4	12	86	14	0
25	29	3	7	41	14	0
35	30	0	8	26	13	0
12	30	3	12	60	15	0
10	26	0	12	53	18	0
15	28	0	6	50	9	0
9	28	0	5	38	7	0
9	28	0	8	32	7	0
15	28	3	10	48	12	0
10	28	2	4	53	10	0
12	26	1	12	68	17	0
10	23	2	8	58	10	0
13	28	0	9	58	13	0
9	28	0	12	77	15	0
12	28	4	11	53	14	0
13	27	1	8	46	14	0
10	23	2	25	78	25	0
9	28	4	8	45	17	0
12	26	0	6	40	15	0
10	22	1	8	56	18	0
10	26	1	8	30	10	0
12	28	0	2	20	4	0
10	22	1	16	80	18	0
13	29	0	2	30	4	0
15	27	4	6	35	10	0
10	28	0	8	45	7	0
12	27	0	5	35	5	0

SUMPD	PD_MEAN	CAL_MEAN	PD14	PD56	PD7	STBOP
195	1,04	0,78	100	0	0	24
283	2,05	2,13	98	2	0	60
387,8	2,38	2,64	85	15	0	138
217	1,8	1,98	97	3	0	21
230,2	1,47	1,51	100	0	0	67
207,8	1,73	1,8	100	0	0	127
240,4	1,42	1,02	100	0	0	4
260	1,6	1,46	100	0	0	13
281,2	1,73	1,65	100	0	0	20
613	3,78	3,81	88	12	0	118
278	1,78	1,83	100	0	0	40
232,2	1,43	1,48	100	0	0	50
494	3,16	3,16	100	0	0	118
305,2	1,81	1,98	100	0	0	32
303,4	1,62	1,64	100	0	0	37
223,2	1,19	1,19	100	0	0	41
199	1,57	1,57	100	0	0	54
309	1,77	1,79	100	0	0	60
271,4	1,62	1,77	100	0	0	47
223,6	1,33	1,32	100	0	0	17
238,8	1,47	1,46	100	0	0	26
408,4	2,43	2,77	97	3	0	79
427,2	2,45	2,45	100	0	0	76
250,2	1,39	1,39	100	0	0	37
509,8	2,83	2,83	100	0	0	89
243,4	1,59	1,35	100	0	0	46
233,8	1,39	1,11	100	0	0	26
247,4	1,47	1,24	100	0	0	21
230,2	1,37	1,02	100	0	0	19
438,2	2,6	2,61	100	0	0	65
356,4	2,12	2,11	92	8	0	67
315,2	2,02	2,02	100	0	0	65
282,8	2,04	2,09	99	1	0	42
276,2	1,64	1,62	100	0	0	35
279,2	1,66	1,66	100	0	0	52
469	2,79	2,79	98	2	0	92
256,2	1,52	1,53	100	0	0	47
322	2,33	2,37	85	15	0	124
270,4	1,69	2,68	98	2	0	68
250,2	1,54	1,54	100	0	0	46
224	1,69	2,1	100	0	0	53
306	1,96	1,97	100	0	0	37
253,4	1,5	1,5	100	0	0	12
283	2,14	2,14	100	0	0	66
248,8	1,42	1,42	100	0	0	12
518,2	3,19	3,32	96	4	0	70
280	1,66	1,66	100	0	0	22
266,2	1,64	1,64	100	0	0	17

CPD	PISA	HAEM1	HAEM2	HAEM3	HAEM4	HAEM5
0	41,159	3	1	1	1	0
52	121,433	0	1	3	1	2
169	506,065	3	1	1	1	3
25	90,325	0	1	0	1	0
0	166,993	0	1	0	1	0
0	300,175	0	4	1	1	2
0	10,031	2	4	1	2	0
0	32,871	3	1	3	1	2
0	54,102	4	4	4	1	4
444	435,271	1	1	1	1	4
8	113,151	1	1	0	1	1
0	144,077	3	1	1	1	0
228	321,312	0	1	0	1	0
4	104,865	3	3	1	3	0
0	107,127	3	1	1	0	0
0	134,899	1	1	3	1	0
4	123,283	0	2	1	1	0
24	142,516	4	1	3	1	0
4	113,433	4	3	1	4	4
0	60,166	1	2	1	1	0
0	55,425	0	2	0	0	0
54	210,82	4	2	3	1	4
60	216,778	1	2	1	1	0
0	100,165	0	3	2	1	0
96	222,995	0	1	0	1	0
0	121,538	0	1	1	1	0
0	55,512	0	1	0	0	0
0	52,096	0	1	0	0	0
0	41,109	0	1	0	0	0
44	209,106	0	1	0	1	0
89	287,024	0	1	0	0	0
4	173,149	1	1	1	0	0
23	105,995	0	1	0	0	1
0	106,073	0	1	1	0	0
0	132,71	1	0	0	0	0
127	277,748	1	1	0	0	0
16	143,86	1	1	0	0	0
101	417,71	0	0	0	0	0
25	70,782	0	0	0	0	0
0	105,472	0	1	0	0	0
24	111,694	0	0	0	0	0
8	94,538	1	1	0	0	0
0	24,508	0	1	0	0	0
28	165,173	0	0	0	0	0
0	32,24	0	1	0	0	0
210	197,083	0	1	0	1	0
0	77	0	0	0	0	0
0	41,736	0	1	0	0	0

HAEM6	HAEM7	HAEM8	HAEM9	HAEM10	HAEM11	HAEM12
0	4	4	1	3	0	0
3	4	4	1	0	0	0
3	4	4	4	4	4	4
0	3	0	0	0	1	0
2	3	4	2	0	0	0
2	3	4	4	3	2	0
0	4	4	0	3	4	1
2	4	4	0	0	1	4
0	4	4	0	0	1	3
0	4	3	0	0	0	3
0	3	3	4	2	0	0
1	4	4	0	0	1	0
0	3	4	3	3	0	0
0	4	0	0	0	3	0
0	2	0	2	3	0	0
0	3	4	0	0	0	0
2	3	4	1	0	1	0
2	4	4	1	0	0	0
2	4	4	4	0	3	4
0	4	0	3	0	0	0
2	4	4	4	3	0	0
3	4	3	2	2	0	0
0	3	4	3	3	0	0
3	3	4	4	0	3	0
2	2	0	0	0	0	0
0	0	0	0	0	0	0
0	0	0	0	0	1	0
2	1	0	0	0	0	1
0	0	0	0	0	0	0
1	0	0	3	0	1	0
0	0	0	0	0	0	0
0	0	0	0	0	0	0
0	1	1	0	0	0	0
0	0	0	0	0	0	0
0	0	0	0	0	0	0
0	0	0	0	0	0	0
0	0	0	0	0	1	0
0	0	0	0	0	0	0
0	0	0	0	0	0	0
0	0	0	0	0	0	0
0	0	0	0	0	0	0
0	0	0	0	0	0	0
0	0	0	0	0	0	0
0	0	0	0	0	0	0
0	0	0	0	0	0	0
0	0	0	1	0	0	0
0	0	0	0	0	0	0
0	0	0	0	0	0	0

HAEM13	HAEM14	VAR_66
0	0	
0	0	
0	4	
0	0	
0	1	
0	2	
0	1	
0	0	
0	0	
0	1	
0	0	
0	1	
0	2	
0	0	
0	0	
0	0	
0	0	
0	0	
0	0	
0	1	
0	0	
0	1	
0	0	
0	1	
0	0	
0	0	
0	0	
0	0	
0	0	
0	0	
0	0	
0	0	
0	0	
0	0	
0	0	
0	0	
0	0	
0	0	
0	0	
0	0	
0	0	
0	0	
0	0	
0	0	
0	0	
0	0	
0	0	
